# Perceived HIV-protective benefits of male circumcision: Risk compensatory behaviour among women in Malawi

**DOI:** 10.1371/journal.pone.0211015

**Published:** 2019-02-27

**Authors:** Blessings Msango Kapumba, Rebecca King

**Affiliations:** 1 Department of Behaviour and Health, Malawi-Liverpool Wellcome Trust, Blantyre, Malawi; 2 Nuffield Centre for International Health and Development, Leeds Institute of Health Sciences, University of Leeds, Leeds, United Kingdom; Weill Cornell Medical College, UNITED STATES

## Abstract

**Background:**

Male circumcision (MC) reduces men’s risk of contracting HIV by approximately 60% and has the potential to significantly alter HIV epidemics. However, MC does not significantly reduce the risk of HIV transmission to women from a circumcised man. In Malawi, several researchers has examined the acceptability, accessibility and sexual behaviour change after circumcision in men but behaviour change in women following their partner’s circumcision remains uncertain. In order to fully realise the protective benefits of MC against HIV, factors related to risky sexual behaviour is imperative as some studies have shown potentials of increased risky behaviour in men following voluntary medical male circumcision (VMMC). This study aimed to explore the perceptions and opinions of female school teachers and health workers on HIV-protective benefits of MC and its impact on risk compensatory behaviour among women in Malawi.

**Methods:**

We conducted a cross-sectional survey of women (N = 68) between May and June 2016 in three districts of southern Malawi. Risk compensatory behaviour was measured by number of sexual partners and use of protection during sex among female teachers and health care workers who are involved with educating people on benefits of VMMC. The bivariable analysis was conducted to test for association between HIV-protective benefits and risk compensatory behaviour. Purposive sampling was used to conduct eight qualitative in-depth interviews with women from the selected districts and the qualitative data was analysed thematically.

**Results:**

The mean age of women who participated in the survey was 30 years. Most women (94.1%) correctly indicated that HIV-positive circumcised men can still infect their partner and approximately, 90% of were knowledgeable of risky sexual behaviour for HIV. However, 55.9% perceived MC can lead women to adopt risky sexual behaviour. On the contrary to this finding, qualitative data indicate women’s misconceptions regarding their partners’ circumcision and HIV-protective benefits. Most women expressed that risky sexual behaviour such as having multiple sexual partners and inconsistent or non-use of condoms can easily be observed among women if they learn of their partners’ partial HIV-protective benefits circumcision.

**Conclusion:**

Exploring women’s sexual behaviour change in the right of HIV-protective benefits of MC fills in a research knowledge important to public health. In-depth studies are therefore required to give more evidence that will guide the development of HIV risk-reduction interventions.

## Introduction

### Male circumcision in Sub-Saharan Africa

Many countries in Sub-Saharan Africa (SSA) including in Malawi which reported HIV prevalence adopted Voluntary Medical Male Circumcision (VMMC) as one strategy for prevention of HIV. VMMC was a recommendation by the WHO in 2007 following significant findings from Randomized Controlled Trials (RCTs) that demonstrated VMMC’s ability to reduce heterosexual transmission of HIV by 60% in men [1–5]. VMMC is now generally accepted in public health interventions and increasingly being accessed by many men in the SSA countries including Malawi [6–8]. Although VMMC have HIV-protective benefits in men, the efficacy remains uncertain in women. Mathematical model on VMMC predicts long-term benefits of decreasing the prevalence of HIV in the population [9]. Such long-term HIV-protective benefits of MC among men extends to women as well, considering that VMMC uptake increases from the current rate [10]. However, sexual behaviour changes in both men and women following VMMC need to be considered in order to make the strategy effective for HIV prevention.

### MC risk compensation for HIV

Risk homeostasis theory suggests that decreases in perceived risk of contracting HIV that occurs with access to HIV prevention technologies, corresponds with increases in risk taking behaviour [11]. RC is the increase in risky behaviour sparked by decrease in perceived risk or adjusted behaviour in response to perceived changes in vulnerability to a disease [11, 12]. HIV prevention technologies such as vaccines, microbicides, antiretroviral medications and other biomedical prevention technologies including medical male circumcision are used in advance of preventing HIV transmission [11]. Risky behaviours associated with vaccines and antiretroviral medications for HIV include high demands for the vaccine and unprotected sex respectively [13]. Similar threats to VMMC programmes in this regard is risk compensation (RC) among women who believe that they are also directly protected from HIV by the circumcision of their partners [14, 15].

Since the adoption of VMMC in several SSA countries including Malawi, researchers have been preoccupied with establishing the level of knowledge, acceptability and up take of VMMC. Notably, RC behaviour have also been explored in men following circumcision. These studies showed little or no RC among men following circumcision, however, studies conducted in Kenya, Zambia, Tanzania and South Africa suggested adoption of RC behaviour among women following circumcision of their male partners [14–17]. Although studies done in Malawi revealed no evidence of change in sexual behaviour among men, this remains hitherto unexplored among women. Behavioural RC has huge implications on HIV prevention strategies, therefore cannot be ignored [12].

While there has been marked improvement in the pace of the scale-up for VMMC, the question for its effectiveness remains; “what impact does VMMC have on behaviour change among women?” Sexual behavioural change such as preference for circumcised men supports the idea that there might be RC among women linked to circumcision status of their partners [17]. Increase in RC behaviour among women may offset the protective benefits of VMMC and defeat the purpose of the strategy.

The study aim to explore perceptions and opinions of female school teachers and health workers who are involved with VMMC campaigns on whether the HIV-protective benefits of MC would influence RC behaviour among women in Malawi. The paper addresses an important gap in the literature “do women adopt riskier sexual behaviours due to perceived HIV-protection of circumcision in men?" It clearly focuses on female secondary school teachers and health worker’s opinions and perceptions of HIV-protective benefits of MC to men and the effects it has on risk compensatory behaviour among women.

## Methods and materials

### Setting

This study was conducted in Zomba, Thyolo and Chiradzulu districts of the Southern of Malawi. These districts were purposively selected because of the long existence of Male Circumcision (MC) performed traditionally as a cultural norm even before the recommendation of VMMC for HIV-prevention [18, 19] and, in-depth information was expected to be gathered from the population. Since the adoption of the VMMC strategy for HIV prevention, these districts were among the first to conduct VMMC campaigns.

### Design

We conducted a cross-sectional survey of women (N = 68) and qualitative in-depth interviews (n = 8) between May and June 2016 in three districts of southern Malawi. The results generated from the survey were triangulated with in-depth responses from qualitative interviews to increase the credibility of the results.

### Sample

#### Study population

The study respondents were female residents of selected districts. The residents consisted a heterogeneous group of health workers and secondary school teachers. Only health care workers and secondary school teachers involved in VMMC services including sexual reproductive health and HIV were eligible to participate. The selection of female secondary school and health workers was based on the fact that they are key stakeholders involved in VMMC campaigns.

#### Sample size and recruitment

The sample comprised of female secondary school teachers and female health workers purposively selected because of their involvement in promotion of VMMC activities. Participants were only considered to be recruited if they met the following criteria: be female, age between 18–45, willingness to participate in the study, able to give informed consent, resident of the selected district, and able to read and write English. The survey sample size for this study was calculated based on the researcher’s interests to measure perceptions and opinions of respondents on risky sexual behaviour among women. The main question to measure this outcome was to “know if respondents understood the partial protection of male circumcision against HIV”, (do you understand how male circumcision reduces the risk of men getting HIV?). Responses to this question were then being associated with perceptions of risk compensation such as condom utilisation and number of casual sexual partners among women. The required sample size was estimated at 71 respondents with a 5% margin of error, a 95% Confidence Interval, and a 10% estimated guess of risk compensation from previous studies [16]. All (n = 71) respondents were recruited in the survey using the above stated inclusion criteria. Participants were recruited at their respective work places. However, only 96% (n = 68) response rate from the self-administered survey questionnaire was achieved.

All qualitative respondents were purposively selected. Female secondary school teachers and health workers were recruited on the basis that they were likely to have in-depth information relevant to address the study objectives [20, 21]. For cost reasons and nature of the study, a sample of eight respondents was considered enough by the researcher to be able to answer the research questions. An appropriate sample size for a qualitative study is one that adequately answers the research question [22]. All respondents were identified and recruited from the survey and they gave informed consent to participate in IDIs as well. For the eight participants, three were from Zomba district (two teachers, one health care worker), three from Thyolo district (two health workers, one teacher), and two from Chiradzulu district (one teacher and one health worker).

#### Interview and survey instruments

Key interview topics were chosen based on a literature review of previous studies related to male circumcision risk compensation, including women’s perceptions and beliefs about male circumcision. Participants were asked questions such as “what do you understand about male circumcision and its HIV protective benefit?”; “Can you explain to me the HIV-protective benefit of male circumcision to women?”; and “what can you explain about change in sexual behaviour in women when they learn that male circumcision provides partial protection to HIV in men?”. The survey included key questions derived from risk sexual behaviour such as condom use, the number of casual sexual partners and women preferences for circumcised men over uncircumcised men. All the data collection tools were pre-tested and necessary changes were made before the actual data collection started.

### Data collection

All survey respondents were approached in person by either a RA or the PI. The recruitment materials were distributed followed by a self-administered survey questionnaire. The respondents were given 72 hours to complete the survey questionnaire which was collected in sealed envelopes by either member of the research team.

Data for both the IDIs and survey was collected simultaneously for the period of three weeks in the month of June, 2016. All eligible respondents were contacted in person by the researcher in all 3 selected districts. All the IDIs were conducted in Chichewa (*local dialect*) using a semi-structured interview guide. All the 8 IDIs were conducted by the recruited female Research Assistant (RA). The IDIs were digitally recorded, and the RA wrote notes that were later expanded. The RA submitted electronic copies of the recording at the end of each day. The first two interviews were listened to by the Principal Investigator (PI) and immediate feedback was given to the RA as part of quality check. All recordings were transcribed verbatim into English by the PI immediately at the end of data collection period. Two RAs assisted in verifying the transcripts by listening to the recordings and reading the transcripts.

### Data analysis

The analysis of data in this study adopted the side-by-side analysis [20]. This analysis approach allows to use qualitative findings enrich themes uncovered by the quantitative analysis [23–25].

Descriptive statistics analysis was conducted for the outcome and control variables by whether or not the respondent understood the partial MC HIV-protective benefits. In order to determine the prevalence of RC, the PI used a Fishers exact test to identify relationship between respondents understanding and perceptions of risk compensation. Fisher’s exact test was preferred because it is recommended for studies with sample size of less than 100 [26]. Data were analysed using Stata (StataCorp.2012. *Stata Statistical Software*: *Release 12*. College Station, TX: StataCorp LP). Relationships were explored between these variables and the perceived women understanding of HIV-protective benefits of male circumcision in order to measure perceptions of risk compensation among women.

The analysis of qualitative transcripts started soon after verification was complete by the RAs. Thematic framework approach following the five steps as described by Gale et al and Ritchie et al was conducted [27, 28]. Accordingly, the framework approach is particularly suited for analysis of cross-sectional descriptive data, allowing different aspects of phenomena under investigation to be captured [28]. The PI read and re-read the transcripts several times to familiarise and gain in-depth understanding of the content. Topics were identified, refined and sorted into index of themes. A working analytical framework was developed after coding the first two transcripts. The codes were organised and grouped into categories relevant to the study objectives. Broader themes were then created from the categories and used to chart the data into a framework matrix on Microsoft excel spreadsheet. All emerging new ideas were categorised as sub-themes.

### Ethics

The study was reviewed and approved by the Leeds Institute of Health Sciences Research Ethics Sub-Committee *(FMHREC-16-2*.*1)* at the University of Leeds and the College of Medicine Research and Ethics Committee (*COMREC*- *P*.*05/06/1958*) in Malawi. Prior to data collection, permission to conduct a study was obtained from all the listed institutions in Appendix 2. Written informed consent was obtained from all study respondents.

## Results

The findings comprise of qualitative and quantitative data from 8 structured interviews and 68 (mean age 30 years) survey responses respectively, Table 1.

**Table 1:** Facilities and institutions where respondents were selected.

District	Facility/institution	Respondent	Number recruited	
			Survey	Interviews
Zomba	Zomba Central Hospital	Female health worker in HIV/STI section	22	2
	Zomba Catholic Secondary School	Female teachers	5	3
	Chinamwali Secondary School	Female teachers	3	1
Chiradzulu	Chiradzulu District Hospital	Female health worker in HIV/STI section	17	
Thyolo	Thyolo District Hospital	Female health worker in HIV/STI section	21	2
Total participants			68	8

Seventy five percent (75%) of the respondents were married and all participants had at least attended up to secondary school and were working either as a teacher or health worker, see Table 2. Several themes emerged from the findings but only those related to the objectives of the study are presented as presented in this section.

**Table 2:** Demographic characteristics of survey and interview respondents.

	Survey respondents (N = 68)	IDIs respondents (N = 8)
**Religion**		
*Christian*	*64(94*.*1%)*	*6(75%)*
*Muslim*	*4(5*.*9%)*	*2(25%)*
**Level of education**		
*Secondary*	*10(14*.*7%)*	*1(12*.*5%)*
*Some college*	*38(55*.*9%)*	*5(62*.*5%)*
*University*	*20(29*.*4%)*	*2(25%)*
**Marital status**		
*Single*	*16(23*.*5%)*	*3(37*.*5%)*
*Married*	*51(75%)*	*5(62*.*5%)*
*Divorced*	*1(1*.*5%)*	
**Occupation**		
*Nurse*	*49(72*.*1%)*	*2(25%)*
*Hospital attendant*	*7(10*.*3%)*	*2(25%)*
*Clinical officer*	*4(5*.*9%)*	
*secondary school teacher*	*6(8*.*8%)*	*4(50%)*
*Others specify*	*2(2*.*9%)*	

### Understanding the HIV-protective benefits of male circumcision

All the survey participants had ever heard of VMMC. A significantly larger proportion of respondents (75%) understood the protective benefit of male circumcision against HIV while 23.5% did not, see Table 3. These findings were related to what respondents described in IDIs where the majority were able to describe male circumcision as a procedure of removing the foreskin of the penis in men. All the respondents had an idea of the reason why VMMC is done and they could relate it very well to reduction in the risk for contracting HIV.

**Table 3:** Summary descriptive analysis of women understanding of MC.

**ever heard of MC**	
*yes*	*68 (100%)*
**understands how MC protects men from getting HIV**	
*yes*	*51 (75%)*
*no*	*16 (23*.*5%)*
*don’t know*	*1 (1*.*5%)*
**circumcised men can still infect their female sexual partners**	
*yes*	*64 (95*.*5%)*
*no*	*1 (1*.*5%)*
*don’t know*	*2 (3%)*
**MC provide full protection against HIV**	
*yes*	*2 (2*.*9%)*
*no*	*65 (95*.*6%)*
*don’t know*	*1 (1*.*5%)*
**women share the HIV-protective benefits**	
*yes*	*45 (66*.*2%)*
*no*	*21 (30*.*9%)*
*don’t know*	*2 (2*.*9%)*
**extent of protection in women**	
*fully protected*	*4 (6%)*
*partially protected*	*51 (76*.*1%)*
*not protected at all*	*9 (13*.*4%)*
*don’t know*	*3 (4*.*5%)*
**women still at risk of HIV infection by a circumcised man**	
*yes*	*65 (95*.*6%)*
*no*	*3 (4*.*4%)*
**rate of the risk**	
*small risk*	*7 (10*.*4%)*
*moderate risk*	*31 (46*.*3%)*
*high risk*	*29 (43*.*3%)*
**women benefit indirectly if their partner is circumcised because a reduction in**	
*True*	*28 (41*.*2%)*
*don’t know*	*18 (26*.*5%)*
*False*	*22 (32*.*4%)*

Respondent’s understanding was also measured with other variables such as, whether HIV-positive circumcised men can still infect their partners, 94.1% sad yes; level of MC protection against HIV in women, 95.6% said it does not; MC only reduces the risk of contracting HIV in both men and women, 79.4% said yes; and MC is beneficial to women, 66.2% said yes and the rest said no. While the general understanding of the HIV-protective benefit of MC among the respondents was high, no significant association was observed on whether the benefit is direct or indirect (OR 1.07; 95% CI: .32–3.59), and *P value 0*.*917*.

Similarly, majority of the respondents in IDIs described the link as being due to the removal of the foreskin of the penis which creates conducive environment for harbouring microorganisms such as HIV virus during sexual intercourse. However, some respondents did not see any protective relationship and argued that MC may even put men at more risk of adopting risky sexual behaviour.

About 75% of respondents indicated that MC provides partial protection against HIV while a small proportion 13% indicated that it provides full protection. An in-depth description of respondents from IDIs had similar views that the protective benefit is partial. However, most of the respondents were mainly referring to protection against cervical cancer in women other than the HIV discussed.

“*The benefit is that it reduces the risk of cancer of cervix…it does not provide 100% protection to HIV*, *40% remains the risk*, *so there is need for using protection…24 years-old_CSS-Z*.”

Most commonly respondents explained partial protection of MC against HIV using phrases like ‘‘reduces the risk of HIV,” provides ‘‘a slight protection” or ‘‘some protection,” and ‘‘does not protect fully.” However, some respondents emphasised that there is no protection without use of extra protection such as condoms.

### Women involvement in MC

Approximately 52% (n = 39) of respondents in the survey indicated that women do get involved in MC activities including discussing with their partners about VMMC, campaigning for VMMC and showing preferences over circumcised men, while the rest did not comment, see Table 4. For those who indicated that women get involved, about 73% considered it to be important. These finding were similar to what respondents described in IDIs. Respondents described such roles as discussing with their partners and fellow women about the benefits of MC, encouraging their partners and son(s) to go for circumcision. Respondents also mentioned that women are very supportive to their partners or son(s) during the healing period after circumcision. Women do this because they are also protected in return.

**Table 4:** Summary descriptive analysis of women involvement on MC.

**women discuss about MC and its HIV-protective benefits in men**	
*yes*	*36 (52*.*9%)*
*no*	*14 (20*.*6%)*
*don’t know*	*18 (26*.*5%)*
	
**decision to make choice to have protected sex in with circumcised men**	
*yes*	*43 (64*.*2%)*
*no*	*18 (26*.*9%)*
*don’t know*	*6 (9%)*

However, some respondents mentioned lack of awareness and in-depth knowledge about the benefits of MC among many women as common factors that hinder their active participation. This would also be another driver for adoption of risk compensatory behaviour.

“*Many women are not aware of MC and do not understand its HIV-protective benefit*. *They think that it is another way that can influence risky sexual behaviours to their partners…28 years-old_FT-CPSC*.”

### Knowledge of risk compensation behaviour for HIV

Most (6) respondents described men as usually the ones who get involved in risky sexual behaviours ending up contracting HIV but due to partial protection offered to them, this applies to women as well. However, the knowledge of reduced risk in women was perceived by some respondents as only possible when the man is HIV negative.

“*MC do protect a woman but only if the partner is HIV-negative and not when already infected…28 years-old_CSS-Z*.”

A about 89.7% of survey respondents indicated that women can still contract HIV from a circumcised man. Most (n = 6) respondents, by contrast, described in IDIs that women are protected even when their partners are HIV positive and circumcised.

“*I still do think that the risk of HIV transmission is reduced to the woman if their partner is circumcised even if their male partner is HIV positive…31 years-old_FT-CPSC*.”

However, within the same group of respondents in IDIs some (n = 2) strongly believed that the risk of contracting HIV remains the same in women in disregard to the circumcision status of their partners.

A large proportion (92.6%) of survey respondents indicated that it is very important for partners to continue using condoms even after circumcision. This was also related to the proportion (97.1%) of respondents who believed that women are still at risk of HIV, see Tables 5 and 6.

**Table 5:** Summary descriptive analysis of knowledge on MC risk compensation.

**A woman can become loose when she learns that her sexual partner is circumcised**	
*yes*	*14 (20*.*6%)*
*no*	*42 (61*.*8%)*
*cannot comment*	*12 (17*.*6%)*
**Women may adopt riskier sexual behaviours**	
*yes*	*22 (32*.*8%)*
*no*	*37 (55*.*2%)*
*don’t know*	*8 (11*.*9%)*
**Men may adopt riskier sexual behaviours**	
*yes*	*42 (62*.*7%)*
*no*	*20 (29*.*9%)*
*don’t know*	*5 (7*.*5%)*
**Women can be forced to have unprotected by circumcised men**	
*yes*	*37 (54*.*4%)*
*no*	*28 (41*.*2%)*
*don’t know*	*3 (4*.*4%)*

**Table 6:** Summary descriptive analysis of current perceptions on risk compensation.

**knowledge of MC HIV protective benefits may lead women to have multiple sexual partners**	
*yes*	*14 (20*.*6%)*
*no*	*47 (69*.*1%)*
*don’t know*	*7 (10*.*3%)*
**couples should still be using protection (condoms) when a partner is circumcised**	
*yes*	*49 (74*.*2%)*
*no*	*12 (18*.*2%)*
*don’t know*	*5 (7*.*6%)*
**significance of continuing using a condom**	
*not that significant*	*5 (7*.*5%)*
*high significance*	*48 (71*.*6%)*
*they wouldn’t mind*	*14 (20*.*9%)*
**MC can lead to women adopting riskier sexual behaviours**	
*yes*	*31 (47%)*
*no*	*32 (48*.*5%)*
*don’t know*	*3 (4*.*5%)*
**condom utilisation may reduce as people learn that MC offers full protection against HIV**	
*yes*	*47 (70*.*1%)*
*no*	*16 (23*.*9%)*
*don’t know*	*4 (6%)*
**riskier sexual behaviours among women can be influenced by the circumcision stat**	
*yes*	*38 (55*.*9%)*
*no*	*27 (39*.*7%)*
*don’t know*	*3 (4*.*4%)*
**the general belief about MC among women about HIV protection**	
*women are also protected fully*	*20 (29*.*9%)*
*women don’t have protection from HIV*	*19 (28*.*4%)*
*male circumcision does not protect from HIV*	*25 (37*.*3%)*
*others thoughts*	*3 (4*.*5%)*
**some women still at risk having unprotected sex with a circumcised man based on**	
*yes*	*60 (88*.*2%)*
*no*	*5 (7*.*4%)*
*don’t know*	*3 (4*.*4%)*
**examples of such groups of women**	
*prostitutes*	*18 (26*.*9%)*
*married women*	*22 (32*.*8%)*
*young girls*	*21 (31*.*3%)*
*others specify*	*6 (9%)*

However, most of the respondents in IDIs stated that women may not mind about the risks of contracting HIV once their partner is circumcised. This belief was very strongly related to the perceived protective benefits of MC against HIV to both partners. However, some respondents thought this usually happen because of lack of understanding of the protective benefits of MC among women. For the majority who said that women may adopt such risky sexual behaviour, also supported the idea that MC provides direct protection to women as well.

“*……some women may start thinking that they are fully protected*. *This would encourage them to have multiple sexual partners with circumcised men believing they are protected…35 years-old_THD-VHC*.”

When asked about examples of risk compensation behaviour, some (n = 5) mentioned having multiple sexual partners and not using condoms. Some respondents also described factors that would lead women to such behaviours as due to unfaithfulness of their partners, lack of satisfaction with one partner and a feeling of more sexual pleasure with circumcised men. Additionally, majority of respondents linked such change in behaviour among women to circumcision of their partner.

“*For those that may adopt risky sexual behaviours I think it’s because of knowledge about the protective-benefits of MC…22 years-old_THD-VHC*.”

### Perceptions of MC risk compensation

Approximately 55.9% of survey respondents viewed MC as a motivating factor for adoption of risky sexual behaviour among women.

**Fig 1:**
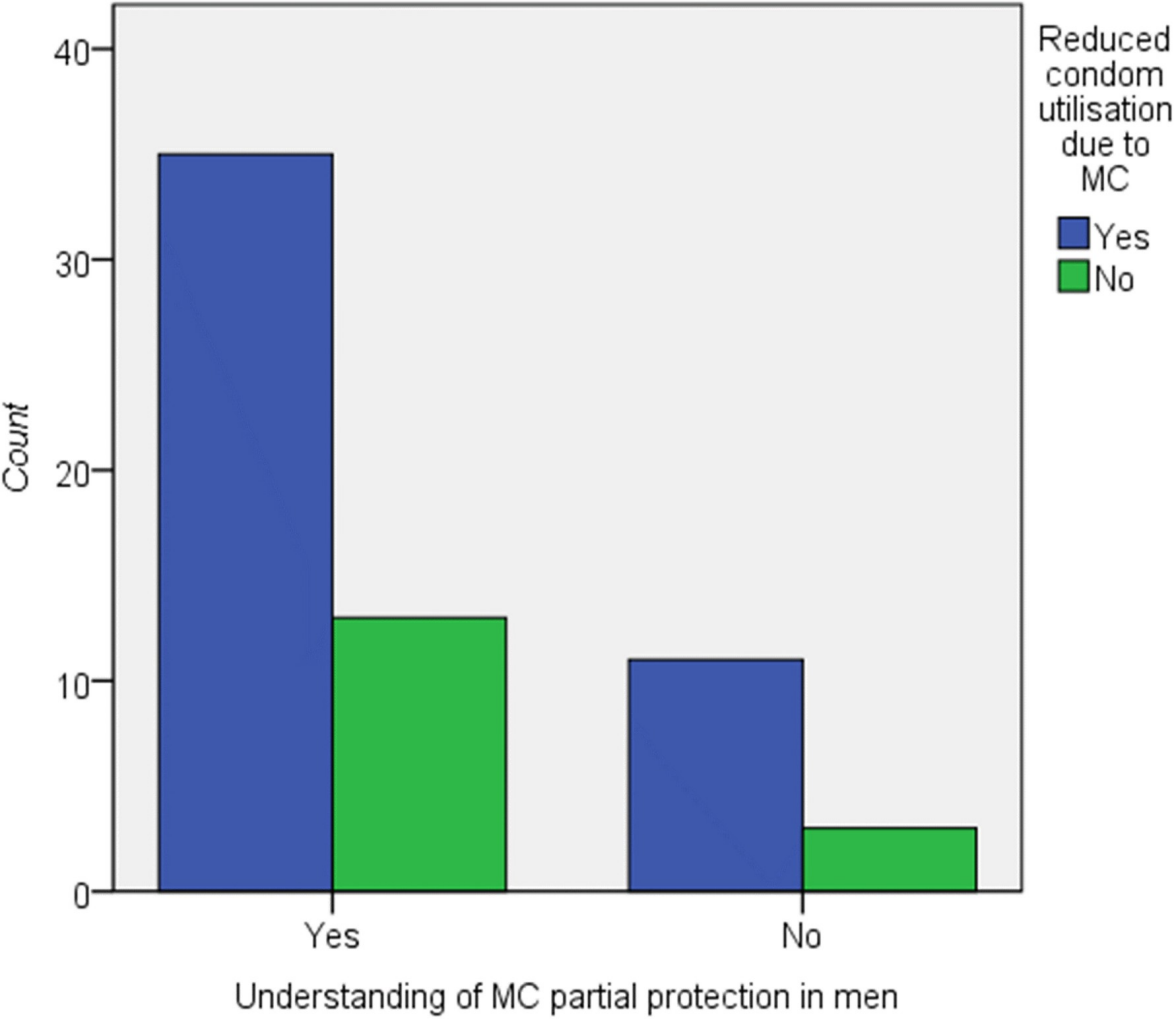
Relationship between understanding of MC and perceptions on reduced condom use for MC. On Fisher’s exact test P value 0.427, p>0.05 showing no significant association between understanding of MC and perceptions on reduced condom use for MC. However, it can evidently be explained from this bar chart that condom utilisation significantly reduces due to knowledge of MC.

**Fig 2:**
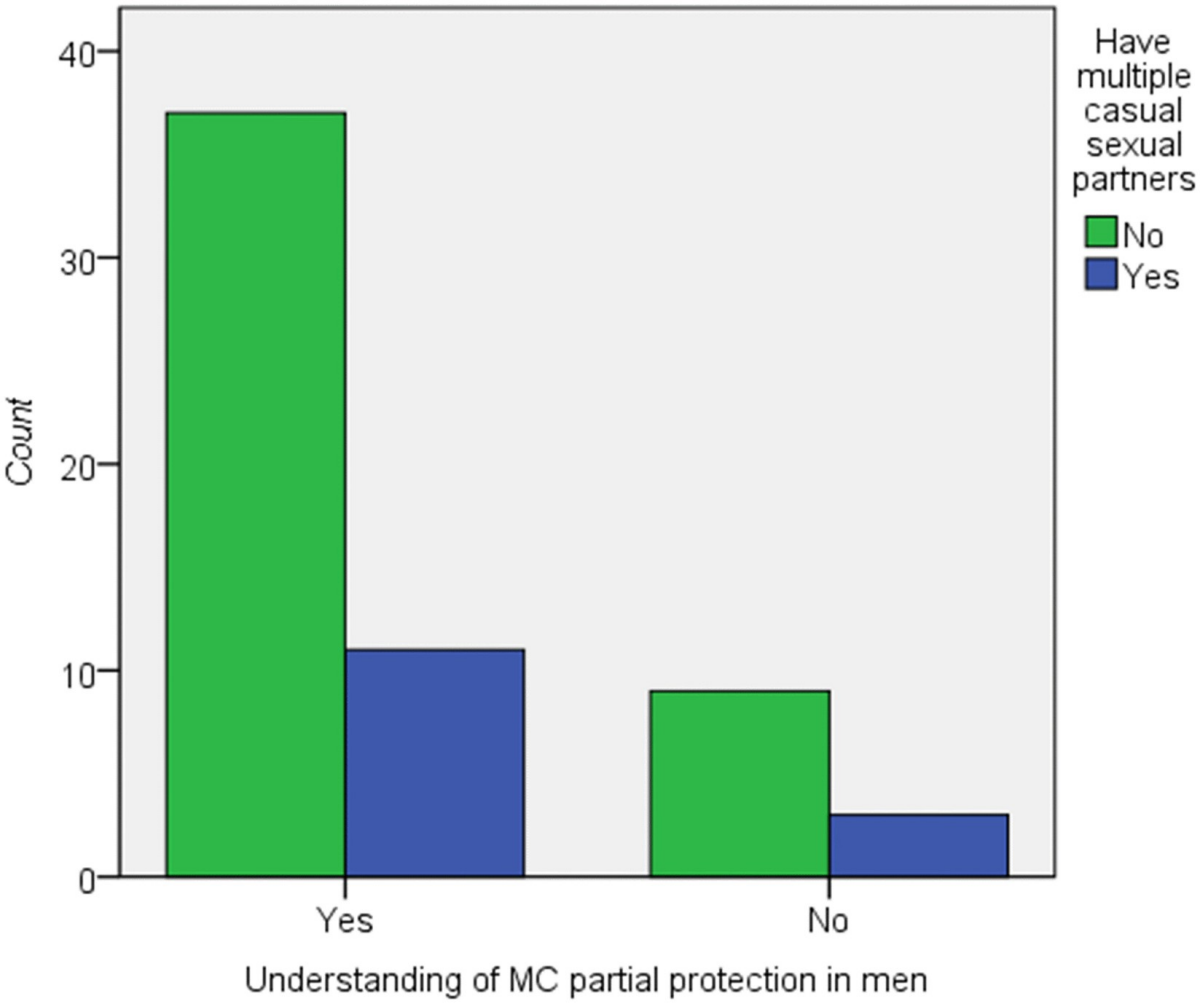
Relationship between understanding of MC and perceptions on having multiple casual sexual partners due to feeling of protected by MC. On Fisher’s exact test P value 0.574, p>0.05 showing no significant association between understanding of MC and perceptions on having multiple sexual partners due to feeling of protected by MC.

However, there was no significant association between understanding of MC and risk compensation factors with a *pvalue = 0*.*574* for likely having multiple sexual partners; *pvalue = 0*.*427* for reduced or non-use of condoms in the population from which the sample data was drawn, Figs 1 and 2.

In IDIs, most of respondents mentioned that MC has influence on current bad sexual practices in Malawi due to the perceived protective benefits against HIV.

“*More people have multiple sexual partners and it is obvious that condom utilisation has dropped…26 years-old_FT-CPSC*.”

However, some respondents also mentioned that as awareness about partial protection increases, condom uptake may substantively increase as well.

Although 61.8% survey respondents did not agree that women may adopt risky sexual behaviour, more than half (61.8%) of the respondents thought that more men would actually adopt risky sexual behaviour in the light of MC. More than half (54.4%) of survey respondents agreed that women could be coerced to have unprotected sex with a circumcised man while 41% disagreed that this could be because of MC, see Tables 4 and 6.

When asked about HIV still being public health threat, majority of IDIs respondents mentioned that people no longer consider HIV as a threat mainly due to availability of medicine that can prolong life. This was supported by some respondents who thought that this also could be one of the driving forces for people to adopt risky sexual behaviours.

“*I don’t think people still regard HIV as a threat to their lives …*., *they know that if infected with HIV*, *they will still be able to live on ARVs…31 years-old_FT-CPSC*.”

There were evidently divergent views from respondents in this study on why women prefer circumcised men. Most of the IDIs respondents explained it is because they believe are safe from HIV, while others it was just for pleasure and satisfaction.

“*Women get attracted to circumcised men*. *…*..*they don’t even know or care about how many partners that person might have*. *Some believe that having sex with a circumcised man gives more pleasure…28 years-old_FT-CPSC*.”

## Discussion

### Understanding MC HIV-protection

The findings from this study indicate that women are aware of the partial HIV-protective benefits of male circumcision. However, there is need to improve women’s understanding as some respondents could not articulate clearly during IDIs the relationship between male circumcision and HIV, and how it does provide protection. While it is true about the HIV-protective benefits, some respondents tended to overstate the protective benefits of MC to both partners. A study by Layer et al in Tanzania showed similar findings where women thought the protection is full [29].

Regarding risk of HIV transmission to women in this era of VMMC, the findings showed a significant need to educate women about the associated risks. A general belief that women are directly protected, and the HIV transmission risk is reduced same as in men was observed in this study which is a threat to VMMC as a strategy for HIV prevention. In a study done by Haberland et al. had similar findings where women had several misconceptions about male circumcison such as believing that it is protective against women too [15]. These misconceptions need to be considered when developing communication tools for HIV risk reduction.

As more respondents from IDIs supported the idea of behaviour change (adopting risky sexual behaviour) among women, findings from the survey did not support that relationship. Generally, lack of in-depth information on male circumcision among women was the main contributing factor and this could be addressed through improved communication in VMMC campaigns. Already, VMMC was supported by the majority in IDIs as being a good strategy for prevention of HIV as recommended by the WHO [1].

### Women involvement in MC

In both the survey and IDIs, findings showed that women play an important role in VMMC activities through discussing with fellow women of its benefits and encouraging their partners and son(s) go for circumcision. Studies conducted in Kenya, Zambia, and Tanzania showed similar findings that women take an active role in advocating for VMMC using means such as refusing to have sex with their partners if uncircumcised [17, 29, 30]. Some suggestions on improving VMMC services such as holding community meeting with women at different forums would be an ideal way of scaling up VMMC programme activities in societies where the uptake is low. Two similar studies which were looking at women involvement had similar findings of considering women being very important in advocating for VMMC to their partners and son(s) [15, 17, 30].

Although there are several advantages of involving women in VMMC activities, information is still limited regarding women involvement in VMMC programmes. There are efforts put in place by the government of Malawi through the MOH to integrate VMMC communication strategies targeting women but the outcome is not very clearly described [7, 31]. These findings also suggest that, women involvement in recommending VMMC to their partners often implies that there is need to seek protection from HIV and cervical cancer. Since no any health programme works in isolation, similarly, VMMC programme may adopt strategies from programmes such as PMTCT and ANC in the arena of involving partners who are not the target of the interventions. However, the findings of this study suggest that women’s involvement in VMMC activities may intentionally or unintentionally influence risky sexual behaviours. This was very clear even in previous studies where women showed preferences for circumcised men [17, 29].

### Knowledge of MC risk compensation behaviour for HIV

There was very little evidence from finding to indicate that women do understood the meaning of risk compensation. Although, respondents were able to articulate some risky sexual practices such as having unprotected sex and multiple casual sexual partners associating with male circumcision and risk compensation for HIV. There is limited information regarding how women understand risk compensation. Although several studies have been conducted in the Southern and Eastern African region using a similar concept, but they don’t show if women understand risk compensation [14–17]. Evidence of some knowledge of risk compensation was articulated by respondents in statements such as perceived protection against HIV extending to women can lead to some women adopting risky sexual behaviour.

### Perceptions on male circumcision and RC for HIV among women

Notably, responses from the survey and IDIs such as reduced or none-utilisation of condoms and increased number of casual sexual partners indicated adoption of risky behaviour. This suggest risk compensation for HIV among women when they learn of the protective benefits of male circumcision. A study conducted in South Africa also revealed similar findings that women were less likely to perceive being at risk of HIV after their partners’ circumcision [16]. This can also be supported by studies from Tanzania where women had strong preference for circumcised men because of the perceived low risk of HIV [29]. Consequently, these findings also showed that women or partners would less likely use condoms and more likely to have unprotected sex with casual partners who have unknown HIV status. This clearly indicates that women perceive reduced or no risk of contracting HIV from a circumcised man. In contrast, these studies did not focus on women experiences when measuring risk compensation.

## Conclusion and limitations

Exploring women’s sexual behaviour change in the right of HIV-protective benefits of MC fills in a research knowledge important to public health. These findings suggest MC risk compensatory behaviour for HIV among women in Malawi and in other countries with similar settings. From a programmatic perspective, it is critical for all MC programmes to reiterate that beliefs about full protection of MC against HIV could potentially minimize its intended benefits. These findings have an impact on the drive to develop risk-reduction interventions. This study had four main limitations. First, the degree of risk compensation was not based on experiences from women with circumcised partners. Therefore, it was difficult to measure the extent to which risk compensation is or is not occurring among women. Second, several other studies have been conducted in the selected districts, higher awareness of MC and partial HIV protection was inevitable, compared to women in other communities where the services are just being scaled up. Third, majority of respondents in this study were married women, recruiting more unmarried women, who are less likely to have regular sexual partners, may have different perceptions of MC, and this may have yielded different results. Finally, findings are from one region of Malawi, they may not be generalizable to other countries that adopted MC in SSA. However, this study provides good basis for a larger study to be conducted across SSA countries.

## Lessons learnt

The concept of risk compensation in women is new in evaluating VMMC programme outcome fitting well to the purpose of this study.

### Further research

The results of this study have relevance for guiding the development of study tools that will be used for a larger study across several countries in the SSA region that adopted MC.

Further exploration of the role of women in effective VMMC program strategies is needed to enable program planners and policy makers to scale up VMMC and achieve maximum HIV reduction in affected communities.

There is need for urgent in-depth studies to undiscover risk compensation among women from their experiences and pragmatically use the finding to design evidence informed HIV risk-reduction interventions.

### Policy

There is need to introduce gender-specific messages in MC programmes highlighting both benefits and limitations of MC for HIV prevention. More emphasis on VMMC campaigns should focus at making explicit that MC does not directly protect women from HIV.

There is need to strengthen integration of MC with other services just like approaches used in PMTCT and ANC in order to increase the MC knowledge base in the population.

## References

[1] WHO and UNAIDS. Joint Strategic Action Framework to Accelerate the Scale-Up of Voluntary Medical Male Circumcision for HIV Prevention in Eastern and Southern Africa (2012–2016). Geneva: UNAIDS 2011.

[2] WHO. New data on male circumcision and HIV prevention: policy and programme implications: WH 2007.

[3] Auvert B., Taljaard D., Lagarde E., Sobngwi-Tambekou J., Sitta R. and Puren A. Randomized, controlled intervention trial of male circumcision for reduction of HIV infection risk: the ANRS 1265 Trial. *PLoS medicine*. 2005, 2(11), p.1112. 10.1371/journal.pmed.0020298 PMC1262556 16231970

[4] Bailey R.C., Moses S., Parker C.B., Agot K., Maclean I., Krieger J.N., et al Male circumcision for HIV prevention in young men in Kisumu, Kenya: a randomised controlled trial. *The Lancet*. 2007, 369(9562), pp.643–656. 10.1016/S0140-6736(07)60312-2 17321310

[5] Gray R.H., Kigozi G., Serwadda D., Makumbi F., Watya S., Nalugoda F., et al Male circumcision for HIV prevention in men in Rakai, Uganda: a randomised trial. *The Lancet*. 2007, 369(9562), pp.657–666. 10.1016/S0140-6736(07)60313-4 17321311

[6] WHO. WHO Progress Brief-Voluntary medical male circumcision for HIV prevention in priority countries of East and Southern Africa. HIV/AIDS 2014.

[7] NAC. *Malawi National HIV Prevention Sstrategy 2015–2020*. Malawi: NAC, 2014.

[8] Westercamp N. and Bailey R. Acceptability of male circumcision for prevention of HIV/AIDS in sub-Saharan Africa: a review. *AIDS and Behavior*. 2007, 11(3), pp.341–355. 10.1007/s10461-006-9169-4 17053855 PMC1847541

[9] Hargrove J., Williams B., Abu-Raddad L., Auvert B., Bollinger L., Dorrington R., Ghani A., et al Male circumcision for HIV prevention in high HIV prevalence settings: what can mathematical modelling contribute to informed decision making? *PLoS medicine*. 2009, 6(9), p.e1000109 10.1371/journal.pmed.1000109 19901974 PMC2731851

[10] Hallett T.B., Alsallaq R.A., Baeten J.M., Weiss H., Celum C., Gray R. et al Will circumcision provide even more protection from HIV to women and men? New estimates of the population impact of circumcision interventions. *Sexually transmitted infections*. 2011, 87(2), pp.88–93. 10.1136/sti.2010.043372 20966458 PMC3272710

[11] Cassell M.M., Halperin D.T., Shelton J.D. and Stanton D. Risk compensation: the Achilles’ heel of innovations in HIV prevention. *BMJ*. 2006, 332(7541), pp.605–607. 10.1136/bmj.332.7541.605 16528088 PMC1397752

[12] Kalichman S., Eaton L. and Pinkerton S. Circumcision for HIV prevention: failure to fully account for behavioral risk compensation. *PLoS Med*. 2007, 4(3), p.e138 10.1371/journal.pmed.0040138 17388676 PMC1831748

[13] Eaton LA, Kalichman SC. Risk compensation in HIV prevention: implications for vaccines, microbicides, and other biomedical HIV prevention technologies. Current hiv/aids Reports. 2007 11 1;4(4):165–72. 18366947 10.1007/s11904-007-0024-7 PMC2937204

[14] Maughan-Brown B., Godlonton S., Thornton R. and Venkataramani A.S. What Do People Actually Learn from Public Health Campaigns? Incorrect Inferences About Male Circumcision and Female HIV Infection Risk Among Men and Women in Malawi. *AIDS and Behavior*. 2014, pp.1–8. 25155700 10.1007/s10461-014-0882-0

[15] Haberland N.A., Kelly C.A., Mulenga D.M., Mensch B.S. and Hewett P.C. Women’s Perceptions and Misperceptions of Male Circumcision: A Mixed Methods Study in Zambia. *PLoS ONE*. 2016, 11(3), p.e0149517 10.1371/journal.pone.0149517 26937971 PMC4777382

[16] Maughan-Brown B. and Venkataramani A.S. Learning that circumcision is protective against HIV: risk compensation among men and women in Cape Town, South Africa. *PLoS ONE*. 2012, 7(7), p.e40753 10.1371/journal.pone.0040753 22829883 PMC3400649

[17] Osaki H., Mshana G., Wambura M., Grund J., Neke N., Kuringe E., et al “If You Are Not Circumcised, I Cannot Say Yes”: The Role of Women in Promoting the Uptake of Voluntary Medical Male Circumcision in Tanzania. *PLoS ONE*. 2015, 10(9), p.e0139009 10.1371/journal.pone.0139009 26402231 PMC4581795

[18] Jung J. Male circumcision pilot program in Lilongwe, Malawi. *Consilience*: *The Journal of Sustainable Development*. 2012, (7), pp.103–114.

[19] WHO. Traditional male circumcision among young people: a public health perspective in the context of HIV prevention. 2009.

[20] Creswell John W. *Research design*: *Qualitative*, *quantitative*, *and mixed methods approaches*. Sage publications, 2013.

[21] Green J. and Thorogood N. *Qualitative methods for health research*. Sage, 2013.

[22] Marshall M.N. Sampling for qualitative research. *Family practice*. 1996, 13(6), pp.522–526. 9023528 10.1093/fampra/13.6.522

[23] Bryman A. Integrating quantitative and qualitative research: how is it done? *Qualitative research*. 2006, 6(1), pp.97–113.

[24] Clark V.L.P. The adoption and practice of mixed methods: US trends in federally funded health-related research. *Qualitative Inquiry*. 2010.

[25] Greene J.C. *Mixed methods in social inquiry*. John Wiley & Sons, 2007.

[26] Pett M.A. *Nonparametric statistics for health care research*: *Statistics for small samples and unusual distributions*. Sage Publications, 2015.

[27] Gale N.K., Heath G., Cameron E., Rashid S. and Redwood S. Using the framework method for the analysis of qualitative data in multi-disciplinary health research. *BMC medical research methodology*. 2013, 13(1), p.117. 24047204 10.1186/1471-2288-13-117 PMC3848812

[28] Ritchie J., Lewis J., Nicholls C.M. and Ormston R. *Qualitative research practice*: *A guide for social science students and researchers*. Sage, 2013.

[29] Layer E.H., Beckham S.W., Mgeni L., Shembilu C., Momburi R.B. and Kennedy C.E. “After my husband’s circumcision, I know that I am safe from diseases”: Women’s Attitudes and Risk Perceptions Towards Male Circumcision in Iringa, Tanzania. *PLoS ONE*. 2013, 8(8), p.e74391 10.1371/journal.pone.0074391 24009771 PMC3756960

[30] Lanham M., L’Engle K.L., Loolpapit M. and Oguma I.O. Women’s roles in voluntary medical male circumcision in Nyanza Province, Kenya. *PLoS ONE*. 2012, 7(9), p.e44825 10.1371/journal.pone.0044825 23028634 PMC3446991

[31] NAC. Communication/Demand Creation, Communication strategies and guidance. [Online]. 2012. [Accessed 03 February, 2016]. https://www.malecircumcision.org/resource/malawi-voluntary-medical-male-circumcision-communication-strategy-2012-2016

